# INTERVENTION DEVELOPMENT: PARTICIPANT EXPERIENCES WITH NOVEL TECHNOLOGICAL DEPRESSION TREATMENTS

**DOI:** 10.1093/geroni/igz038.1663

**Published:** 2019-11-08

**Authors:** Avani A Shah, Martin Morthland, Forrest Scogin, Andrew Presnell

**Affiliations:** University of Alabama, Tuscaloosa, Alabama, United States

## Abstract

The purpose of this presentation is to discuss the development process of two novel technology-based interventions for depression in older adults while comparing older adults’ preferences for audio-based and computer-based cognitive behavioral therapy for depressive symptoms. The audio program consisted of eight compact discs and a workbook while the computer program consisted of 11 modules of similar duration provided on a tablet PC. Fifty-one older adults were recruited from medical settings and rural communities and randomly assigned to an immediate treatment group (computer or audio) with minimal contact or a four-week minimal contact delayed treatment control condition. Participants rated computer-based and audio-based cognitive behavioral therapy fairly equally, with 75% of those who received audio treatment and 85% of those who received computer-based treatment indicating benefits to their mood. Qualitative experiences will be presented by themes: challenges, benefits, recommended improvements, and technological issues. The goal of the presentation is to share information that may help clinicians and researchers develop other mental health technological interventions tailored for older adults needs.

